# Immediate postoperative non‐invasive positive pressure ventilation following midface microvascular free flap reconstruction

**DOI:** 10.1002/cnr2.1518

**Published:** 2021-10-27

**Authors:** Andrew R. Larson, Jenny X. Chen, Allison Holman, Stacey Sullivan, Purris Williams, Katharine Nicholson, Derrick T. Lin, Yuka Kiyota, Jeremy D. Richmon

**Affiliations:** ^1^ Department of Otolaryngology – Head and Neck Surgery Massachusetts Eye and Ear, Harvard Medical School Boston Massachusetts USA; ^2^ Department of Speech, Language, and Swallowing Disorders Massachusetts General Hospital Boston Massachusetts USA; ^3^ Sean M. Healy & AMG Center for ALS, Department of Neurology Massachusetts General Hospital Boston Massachusetts USA; ^4^ Department of Anesthesiology Massachusetts Eye and Ear Boston Massachusetts USA

**Keywords:** amyotrophic lateral sclerosis, head and neck cancer, head and neck reconstruction, microvascular reconstruction, positive pressure ventilation

## Abstract

**Background:**

There is a rare need for postoperative non‐invasive positive pressure ventilation (NIPPV) following microvascular reconstruction of the head and neck. In midface reconstruction, the free flap vascular pedicle is especially vulnerable to the compressive forces of positive pressure delivery.

**Case:**

A 60 year old female with Amyotrophic Lateral Sclerosis (ALS) presented with squamous cell carcinoma of the anterior maxilla, for which she underwent infrastructure maxillectomy and fibula free flap reconstruction. To avoid tracheotomy, the patient was extubated postoperatively and transitioned to NIPPV immediately utilizing a full‐face positive pressure mask with a soft and flexible sealing layer. The patient was successfully transitioned to NIPPV immediately after extubation. The free flap exhibited no signs of vascular compromise postoperatively, and healed very well.

**Conclusion:**

Postoperative non‐invasive positive pressure ventilation can be successfully applied following complex microvascular midface reconstruction to avoid tracheotomy in select patients without vascular compromise of the free flap.

## 1 INTRODUCTION

There has been increasing evidence that tracheotomy is not required for a subset of head and neck cancer patients who undergo free flap reconstruction.^1^, ^2^, ^3^ Avoidance of tracheotomy simplifies postoperative recovery, allows for early post‐operative phonation, avoids complications of tracheotomy, and potentially shortens the postoperative hospital stay.^3^, ^4^, ^5^ However, decision‐making regarding perioperative tracheotomy is multifactorial and must incorporate the anticipated volume of the ablative defect and reconstruction, expected postoperative swelling, and underlying patient morbidities and body habitus.

In particular, amyotrophic lateral sclerosis (ALS) poses challenges for airway management following free flap head and neck cancer reconstruction. ALS is a neuromuscular disease of the upper and lower motor neurons that causes progressive respiratory insufficiency.^6^, ^7^ Tracheotomy in ALS patients is generally reserved for end‐stage disease and should be avoided unless absolutely necessary given known difficulty with decannulation. This imperative is further complicated by the fact that ALS patients underoing anesthesia in general have a high risk of prolonged postoperative intubation and hospitalization after surgical procedures.^8^, ^9^

Non‐invasive positive pressure ventilation (NIPPV) is frequently used for ALS patients to faciliate transition off the ventilator following extubation from general anesthesia. For the reconstructive head and neck surgeon, NIPPV poses a risk to the microvascular pedicle; as such, pedicle location and geometry are critical factors to consider when expecting to apply NIPPV postoperatively. Furthermore, NIPPV is frequently used as an important adjunct for ALS patients in general to improve quality of life and life expectancy.^10^, ^11^, ^12^ Thus, the treating surgeon must be prepared to navigate use of a NIPPV system around the microvascular reconstruction.

Here we highlight a clinical case of midface microvascular reconstruction in an ALS patient with squamous cell carcinoma of the anterior maxilla to demonstrate techniques for protecting the microvascular reconstruction when tracheotomy should be avoided and NIPPV is required postoperatively.

## 2 CASE 1

Written, informed consent for the publication of case details and identifying photographs and radiographs was provided by the patient.

### 2.1 Clinical Presentation

**FIGURE 1:**
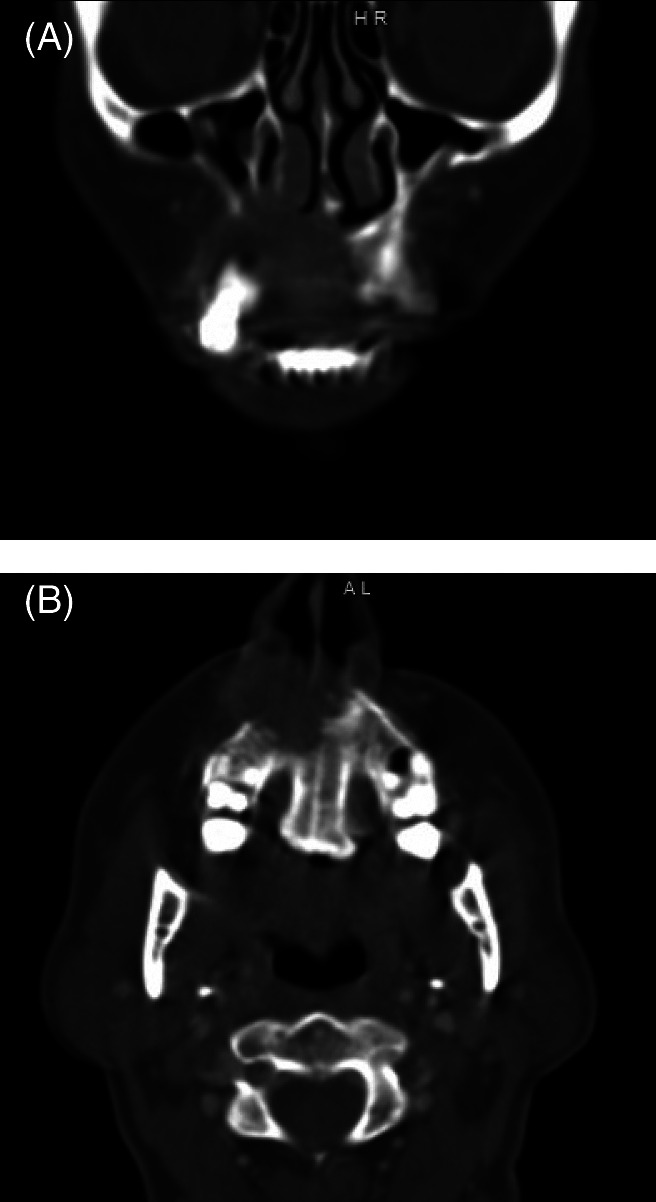
Representative preoperative coronal (A) and axial (B) computerized tomography slices of the tumor of the anterior maxilla crossing midline

A 60 year old female presented to Head and Neck Clinic with a 3‐4 month history of a lesion of the anterior alveolar ridge. Biopsies revealed invasive squamous cell carcinoma (SCC). Clinically, she had a 4 cm lesion of the anterior maxilla, and radiographically the tumor eroded just through the floor of the nasal cavity but did not approach the orbit (Figure 1). Her case was discussed at multidisciplinary tumor board, with consensus recommendation for upfront surgery via infrastructure maxillectomy.

The patient was diagnosed with slowly progressive limb‐onset familial ALS 3 years prior to this cancer diagnosis, with subsequent onset of respiratory and bulbar symptoms. At the time of presentation, she was wheelchair‐bound, partially gastrostomy tube dependent (tolerating only limited pureed food by mouth), requiring symptom management for sialorrhea, and using NIPPV at night for orthopnea with normal speech function. Pre‐operative pulmonary function testing showed a vital capacity of 51% of predicted normal. Given the underlying respiratory insufficiency due to her ALS, there was concern that the patient would be difficult to decannulate should she undergo tracheotomy perioperatively, and that by doing so she would be prematurely committed to lifetime tracheotomy dependence. The patient was admitted pre‐operatively to optimize her overall status and evaluate if her NIPPV face mask could be incorporated post‐operatively. Multi‐disciplinary input between the surgical team, neurology, respiratory therapy, anesthesia, speech‐language pathology, and patient resulted in a consensus decision to attempt to avoid a tracheotomy.

### 2.2 Surgical Details

**FIGURE 2:**
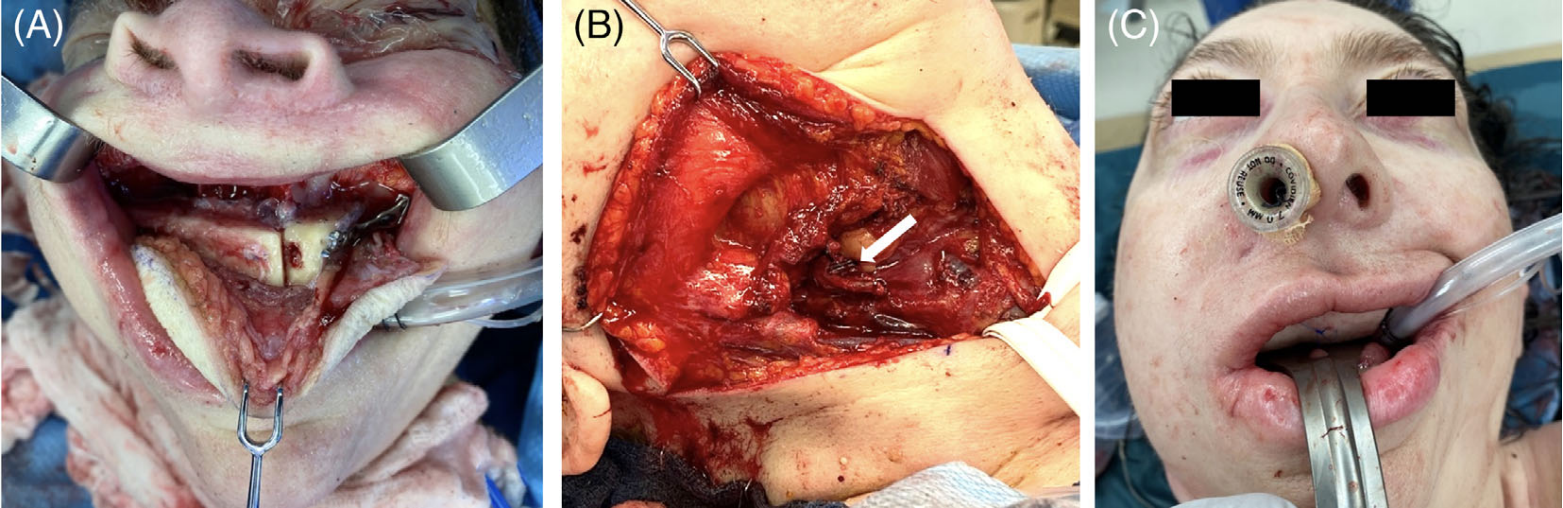
Intraoperative photos of two‐segment osteocutaneous fibula free flap midface reconstruction (A); free flap pedicle tunneled medial to mandible (arrow) prior to microvascular anastomosis with the facial artery and vein (B); and postoperative appearance of the patient's midface and well‐vascularized free flap skin paddle at the finalization of the case (C)

The patient underwent an uncomplicated infrastructure maxillectomy, ipsilateral neck dissection, and fibula free flap reconstruction via a two‐surgeon synchronous approach to minimize operative time. Two‐segment vascularized bony reconstruction was utilized to reconstruct the anterior maxilla (Figure 2). The 5 × 10 cm fibula skin paddle was utilized for reconstruction of palatal, alveolar, buccal, and lip mucosa. The free flap pedicle was tunneled submucosally along the retromolar trigone, and medial to the mandible into the neck for microvascular anastomosis to the right facial artery and vein (Figure 2). A 3.0 mm venous coupler was utilized for the venous anastomosis. Total anesthesia time was 448 minutes.

### 2.3 Outcomes

**FIGURE 3:**
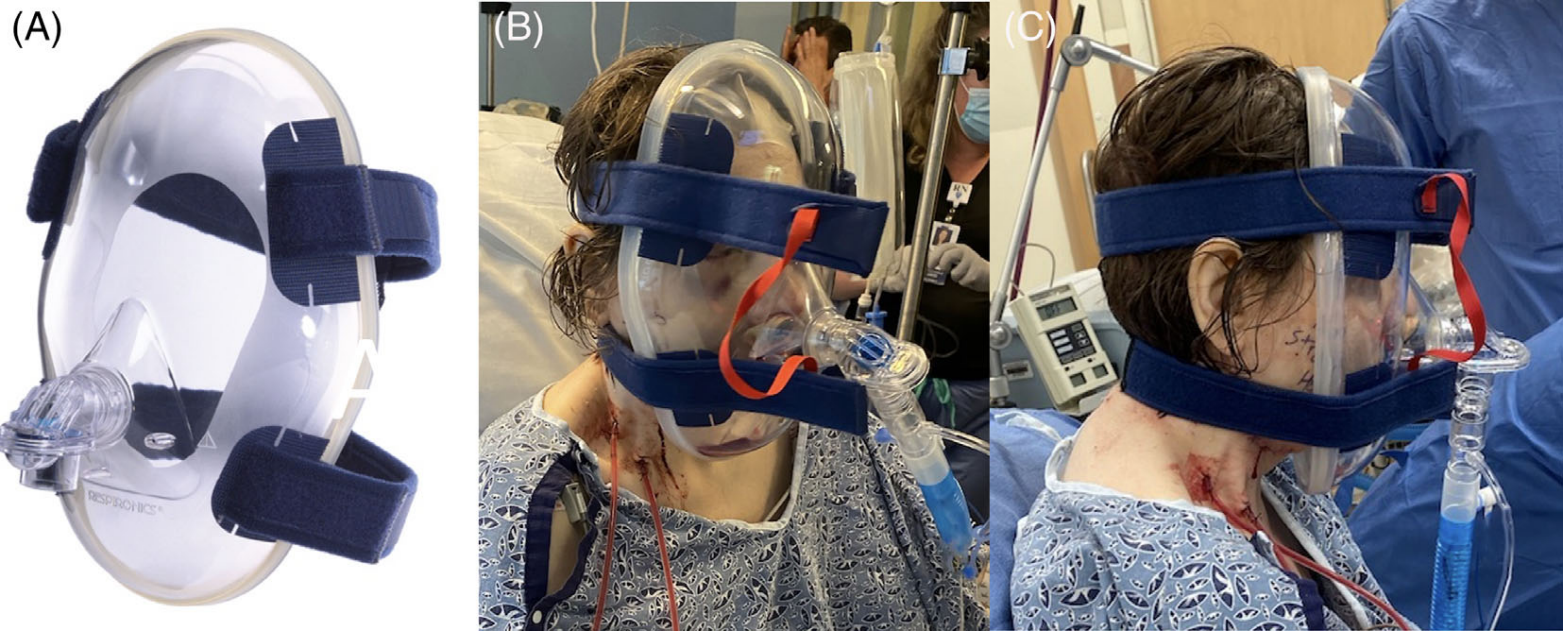
Philips Respironics Total Face Mask with a soft, flexible sealing layer [photo from product brochure, 2005] (A) applied immediately postoperatively (B), (C) for BiPAP delivery following maxillectomy, neck dissection, and osteocuteanous fibula free flap reconstruction of the midface

Immediately following extubation, the patient was transitioned to NIPPV pulmonary support with a Philips Respironics Total Face Mask (Murrysville, PA; Figure 3). Following transfer into the intensive care unit, the patient was weaned from NIPPV to supplemental oxygen via facemask within 4 hour of transfer to the ICU, without need for positive pressure ventilation for the remainder of her inpatient stay.

**FIGURE 4:**
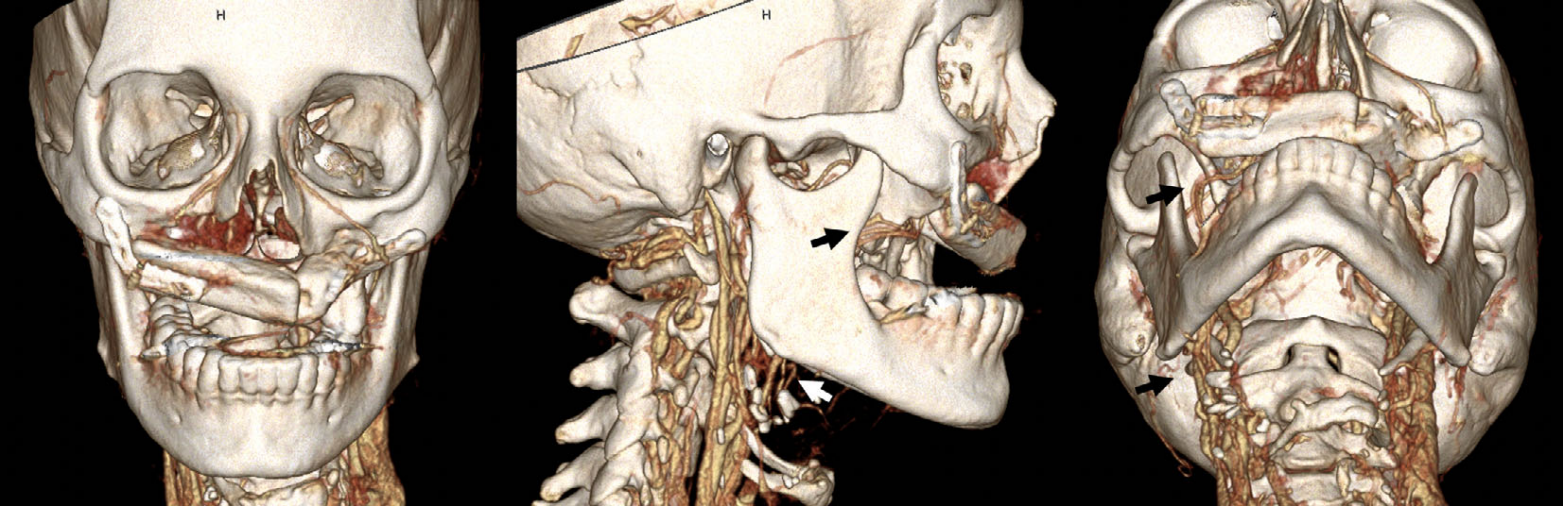
3D Reconstruction of postoperative CT scan demonstrating fibula free flap midface reconstruction with vascular pedicle (arrows) running medial to mandible

Throughout her immediate postoperative course, the free flap maintained excellent perfusion as evidenced by brisk capillary refill, appropriate color, and strong biphasic doppler signal on the skin paddle. There were no signs of venous congestion or partial or full skin paddle loss noted. At the time of outpatient follow up (POD 24), the skin paddle was well healed with all intraoral incisions intact, and the anterior maxilla displayed appropriate projection (see Figure 4). The patient participated in a video swallow study on POD 24, revealing safe and efficient swallowing which allowed resumption of her pre‐surgical diet of pureed foods and nectar thick liquids for pleasure. However, she remained partially G‐tube dependent given pre‐existing neuromuscular dysphagia.

## 3 DISCUSSION

Here we describe the novel application of immediate postoperative NIPPV of a patient with ALS without vascular compromise of midface fibula free flap reconstruction. Key factors to success of the reconstruction included a medial tunnel of the vascular pedicle along the inner aspect of the mandible, as well as use of a full face NIPPV mask with a soft sealing layer which did not place direct pressure on the midface reconstruction.

Typically, the tunnel for the pedicle for midfacial reconstruction with the fibula free flap is created subcutaneously, passing lateral to the mandible.^13^, ^14^, ^15^ Here we describe alternatively creating the tunnel medial the mandible in order to protect the pedicle from compression of the NIPPV system. The submucosal dissection was straightforward, without inadvertant mucosal tear during the dissection. In addition, the patient was placed on prophylactic steroids postoperatively, and no significant edema of the oral cavity or oropharynx was noted as a result of the submucosal intraoral tunnel. However, adequate length of the pedicle for this technique must be ensured in order to avoid a difficult microvascular anastomosis near or underneath the mandible.

This case also highlights important considerations when treating ALS patients with head and neck cancer. First, the surgeon must carefully weigh the donor site morbidity when considering free flap reconstruction as there may be a discrepancy in function between the upper and lower extremities depending on the patient's neurologic status. In this case, given that the patient had no residual function in her lower extremities, a fibula free flap posed minimal donor site morbidity.

The head and surgeon should carefully counsel a patient with ALS that decannulation following tracheotomy for a non‐pulmonary purpose (eg, airway protection following head and neck reconstruction) may be prolonged and/or more difficult than the typical head and neck cancer patient. In this case, a tracheotomy was avoided by transitioning the patient directly from the ventilator to NIPPV as she recovered from general anesthesia, and we encountered no issues with airway compromise postoperatively. Additionally, it allowed for immediate oral speech which was her perferred mode of communication in the setting of declining function of her upper extremities. Patients with ALS ultimately lose the ability to phonate and rely on assistive modes of communication, which may be impacted by surgery. Ultimately, multidisciplinary discussions between the surgeon, pulmonologist, anesthesiologist, neurologist, respiratory therapist, speech‐language pathologist, and nursing staff are critical for difficult perioperative decision‐making for these patients.

In non‐ALS setting, tracheotomy can often be avoided in free flap reconstruction of the palate, particularly in the scenario of a patient with robust physiologic reserve and a modest‐sized intraoral reconstruction. However, in patients with ALS with poor pulmonary reserve and impaired secretion management, airway management after any intraoral reconstruction is inevitably more complex. A non‐ALS patient can often compensate for the increased secretion burden and swelling associated with such a palatal reconstruction. However, this manuscript highlights the adjunctive measures which can help bridge the patient though the perioperative period to compensate for the airway insults necessarily inflicted by the oral cavity reconstruction, and thus avoid perioperative tracheotomy. In addition, we foresee these methods of post‐operative NIPPV in the context of palatal free flap reconstruction could potentially be applied to the free flap reconstruction of other oral cavity defects for which elective tracheotomy could be avoided in a non‐ALS patient, for example, buccal mucosa or anterior tongue/floor of mouth without significant detachment of geniohyoid or genioglossus musculature.

Lastly, when treating patients with ALS, it is paramount for the head and neck surgeon to incorporate the larger picture of the patient's life expectancy and quality of life goals into surgical decision‐making. Any surgical treatment, even in the case of head and neck cancer, is necessarily palliative in this progressive neurologic disease which is universally fatal.^16^ Incorporating the treating neurologist's predictions of life expectancy, the speech‐language pathologist's expectations for speech and swallowing outcomes, as well as potentially including a palliative care physician in discussion of goals of care preoperatively, are important aspects when approaching surgical cancer care of ALS patients.

## 4 CONCLUSIONS

We describe a successful system for immediate postoperative NIPPV for a patient with ALS following midface reconstruction with a fibula free flap, with excellent healing without vascular compromise of the free flap postoperatively. Keys to avoidance of pedicle or free flap compression by the NIPPV mask in this case included a medial tunnel for the free flap pedicle along the inner aspect of the mandible, as well as use of a full face NIPPV mask with a soft sealing skin interface that did not cause direct pressure to the free flap. This case also highlights important perioperative considerations for the head and neck surgeon when treating ALS patients, including extremity function when considering the donor site, as well as concerns for difficulty with decannulation following tracheotomy.

## CONFLICT OF INTERESTS

The authors certify that they have no affiliations with or involvement in any organization or entity with any financial interest or nonfinancial interest in the subject matter or materials discussed in this manuscript.

## ETHICAL STATEMENT

Written, informed consent was obtained directly from the patient for publication of this case and all photos included therein. Given that this manuscript includes the description of one single case, it does not meet criteria for “human subjects research” and as such is exempt from IRB review.

## AUTHOR CONTRIBUTIONS

*Conceptualization*, A.R.L., J.D.R.; *Investigation*, A.R.L., J.D.R.; *Writing‐original draft*, A.R.L.; *Writing‐review & editing*, A.R.L., J.D.R.; *Supervision*, J.D.R.; *Validation*, A.R.L.; *Project administration*, J.D.R.; *Data curation*, A.R.L.

## Data Availability

The data that support the findings of this study are available from the corresponding author upon reasonable request.
